# Impact of Deep Learning-Based Image Reconstruction on Tumor Visibility and Diagnostic Confidence in Computed Tomography

**DOI:** 10.3390/bioengineering11121285

**Published:** 2024-12-18

**Authors:** Marie Bertl, Friedrich-Georg Hahne, Stephanie Gräger, Andreas Heinrich

**Affiliations:** Department of Radiology, Jena University Hospital, Friedrich Schiller University, 07747 Jena, Germany

**Keywords:** artificial intelligence, computed tomography, deep learning, image reconstruction, tumor staging

## Abstract

Deep learning image reconstruction (DLIR) has shown potential to enhance computed tomography (CT) image quality, but its impact on tumor visibility and adoption among radiologists with varying experience levels remains unclear. This study compared the performance of two deep learning-based image reconstruction methods, DLIR and Pixelshine, an adaptive statistical iterative reconstruction—volume (ASIR-V) method, and filtered back projection (FBP) across 33 contrast-enhanced CT staging examinations, evaluated by 20–24 radiologists. The signal-to-noise ratio (SNR) and contrast-to-noise ratio (CNR) were measured for tumor and surrounding organ tissues across DLIR (Low, Medium, High), Pixelshine (Soft, Ultrasoft), ASIR-V (30–100%), and FBP. In two blinded surveys, radiologists ranked eight reconstructions and assessed four using a 5-point Likert scale in arterial and portal venous phases. DLIR consistently outperformed other methods in SNR, CNR, image quality, image interpretation, structural differentiability and diagnostic certainty. Pixelshine performed comparably only to ASIR-V 50%. No significant differences were observed between junior and senior radiologists. In conclusion, DLIR-based techniques have the potential to establish a new benchmark in clinical CT imaging, offering superior image quality for tumor staging, enhanced diagnostic capabilities, and seamless integration into existing workflows without requiring an extensive learning curve.

## 1. Introduction

Computed tomography (CT) reconstruction techniques have evolved from filtered back projection (FBP) to iterative reconstruction (IR), and with the advent of deep learning (DL), are now entering a new era. DL applications, such as TrueFidelity deep learning image reconstruction (DLIR) by GE Healthcare [1] and Pixelshine (PS) by AlgoMedica [2], have shown significant improvements in noise reduction, sharpness, signal-to-noise ratio (SNR), contrast-to-noise ratio (CNR), artifact minimization, and radiation dose reduction [3,4,5,6,7,8,9]. Additionally, DL addresses the issue found with IR-based images, which can sometimes appear unnatural and plastic-like [10,11,12] and are often rejected by radiologists. Despite the progress, most studies [3,4,5,6,7,8,9] to date have focused predominantly on image quality and dose reduction, with limited emphasis on the diagnostic performance. Additionally, qualitative assessments are often limited to evaluations by only one or two experienced radiologists and generally do not encompass multiple contrast phases or direct comparisons of different DL techniques. Further research is needed [13] to assess potential diagnostic limitations, such as the impact of blurring on small structures.

This study aims to address these gaps by comparing the diagnostic evaluation of tumor findings in contrast-enhanced CT images reconstructed using a variety of techniques, including advanced DL-based methods. By involving 20–24 radiologists with varying levels of expertise, the study provides a comprehensive analysis of their preferences and potential biases. Furthermore, by examining the same clinical findings across both arterial and portal venous phases, the study seeks to identify practical differences in image interpretation, structural differentiability, and diagnostic certainty, with the goal of evaluating the potential of DL technology to enhance diagnostic performance in diverse clinical scenarios.

## 2. Materials and Methods

All methods used in the study have been approved by the local ethics committee of the Jena University Hospital (registration number 2020-1725-Daten) and were carried out in accordance with relevant guidelines and regulations. As this was a retrospective analysis of routine work, written informed consent was waived by the ethics committee.

### 2.1. Study Population

**Figure 1:**
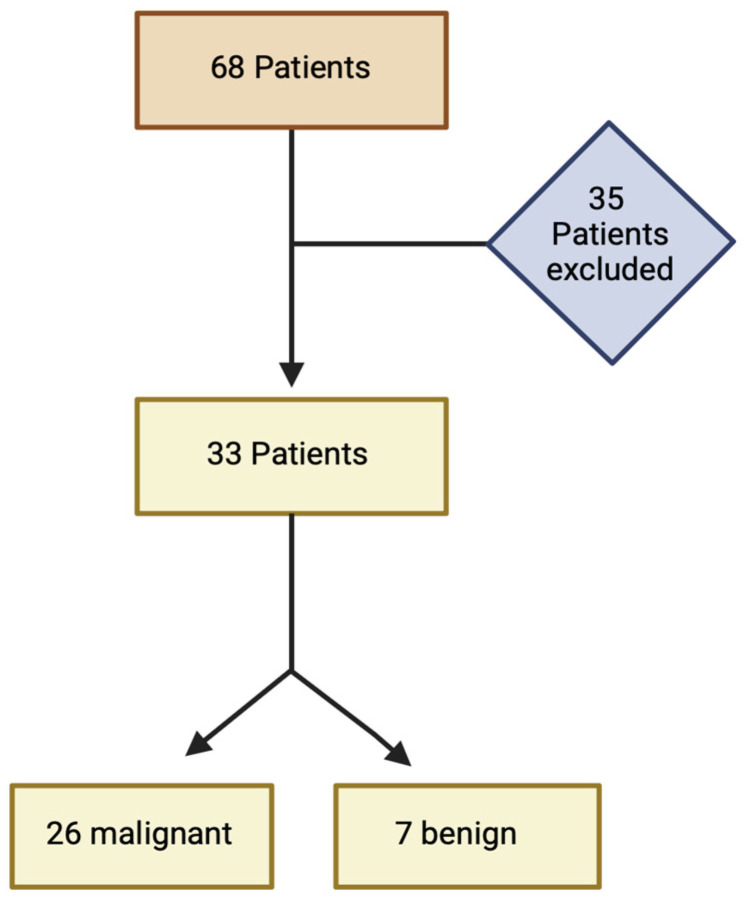
Flowchart of the study population.

In this retrospective study, 68 contrast-enhanced routine CT staging examinations were analyzed. Patients were included based on the criterion of having a detectable finding in both the portal venous and arterial phase. This inclusion criterion was met by 33 examinations, comprising 26 malignant and 7 benign tumor findings, as shown in Figure 1.

The most common tumor types identified were liver (10 cases), kidney (4), lymph nodes (3), and lung (3), along with smaller counts of other tumors, including pancreas, stomach, esophagus (2 each). Less common types included gallbladder, heart, duodenum, lymphocele, abdominal wall metastasis, breast, and spleen (one each). The patient data (Table 1) included a mean age of 64.09 ± 12.77 years, a body mass index (BMI) of 27.49 ± 6.13, with 17 females and 16 males.

### 2.2. CT Data Acquisition

All patients included in this study underwent routine CT examinations using a 256-slice multi-detector CT scanner (Revolution CT; GE Healthcare Technologies, Waukesha, WI, USA) following a standardized staging protocol. Imaging was performed in the native, arterial, and portal venous phases. Tube voltage was 120 kVp in most cases but occasionally 100 kVp, with mean values of 117.6 ± 6.63 kVp (native), 113.9 ± 9.33 kVp (arterial), and 112.7 ± 9.77 kVp (portal venous). The tube current averaged 211.5 ± 99.6 mA, 199.1 ± 76.5 mA, and 261.0 ± 117.7 mA, respectively. All scans were performed in helical mode with a consistent pitch factor of 0.99, a slice thickness of 0.625 mm, and a 512 × 512 reconstruction matrix for all phases. A contrast medium (60 ± 10 mL) was administered intravenously through the antecubital vein. Arterial phase scanning was triggered using bolus tracking (SmartPrep; GE Healthcare) at the level of the aortic root. Volumetric CT dose indices (CTDI_vol_) and dose length product (DLP) were recorded for each acquisition from the dose management system.

### 2.3. Image Reconstructions

The same CT raw data were processed using ten different reconstruction algorithms. These algorithms can be categorized into three main types: DL-based reconstructions such as DLIR at the strength levels of Low, Medium, and High, as well as PS (Version 1.2) at the strength levels of Soft and Ultrasoft. Additionally, IR-based methods were utilized, including adaptive statistical iterative reconstruction—volume (ASIR-V) with blending factors of 30%, 50%, 70% and 100%, alongside the standard technique FBP. ASIR-V, functioning as a hybrid IR algorithm, generates blended images between FBP and IR, incorporating IR percentages ranging from 0% (FBP) to 100% (pure IR image). The reconstruction parameters were inherently determined by the predefined strength levels of each method, with no additional adjustments possible. All reconstructions were performed using the same CT raw data and the standard reconstruction kernel, ensuring consistency and comparability across methods.

#### 2.3.1. TrueFidelity

TrueFidelity DLIR [1] by GE Healthcare, introduced in 2019, is a commercially available technology exclusively designed for GE CT scanners. This technology was trained using high-quality FBP images at high doses, which serve as a reference model for a Deep Neural Network (DNN). Through iterative training with differential analysis between low-dose and high-dose images, the model was optimized and stabilized to maintain image quality at reduced doses. The resulting TrueFidelity CT images resemble high-dose FBP images but are generated at lower doses. DLIR offers three available strength levels (Low, Medium, High) that allow for flexible adjustment of noise reduction to meet specific requirements and applications in various clinical scenarios. It is particularly suitable for high-resolution applications and provides consistent results regardless of the applied radiation dose or image contrast [1,14,15]. However, the technology is exclusive to GE Healthcare CT scanners and is not universally applicable.

#### 2.3.2. Pixelshine

PS by AlgoMedica [2] was introduced in 2019 and is a commercially available DL-based software for noise reduction and image enhancement in CT scans. The software operates on a separate, independent server that receives Digital Imaging and Communications in Medicine (DICOM) files, optimizes them, and sends them back to the original system. This makes PS compatible with CT devices from various manufacturers, offering universal applicability. PS provides different strength levels like Soft and Ultrasoft to adjust the degree of noise reduction according to varying clinical needs.

### 2.4. Quantitative Evaluation

For the quantitative analysis, the SNR and CNR were calculated for the native, arterial, and portal venous phases of the 33 tumor findings for all reconstructions. Two regions of interest (ROIs) were placed, one in the tumor (mean area: 269 pixels) and one in the surrounding organ tissue (mean area: 1474 pixels). Custom software using QML and the Qt framework (version 5.1) was used to measure the mean signal (S) and standard deviation (SD) within both ROIs across all image reconstructions, with the ROIs consistently placed at the exact same location. The SNR was calculated for each finding using the following formula:

SNR = S_tissue_/SD_tissue_ (1)

Due to the small ROI size caused by the tumor’s size, the SNR was measured in the directly surrounding tissue for greater measurement accuracy. The CNR was calculated to quantify the contrast between the tumor and the directly surrounding tissue using the following formula:

CNR = |S_tissue_ − S_tumor_|/SD_tissue_ (2)

### 2.5. Qualitative Evaluation

The qualitative evaluation was divided into two distinct parts: a ranking from best to worst based on the evaluation of overall image quality and a Likert scale assessment to evaluate specific diagnostic attributes. A total of 28 radiologists participated in these evaluations. However, due to varying participation, 20 radiologists completed the first part, while 24 completed the second. The group composition varied slightly between surveys, as some radiologists participated in only one.

#### 2.5.1. First Part

**Figure 2:**
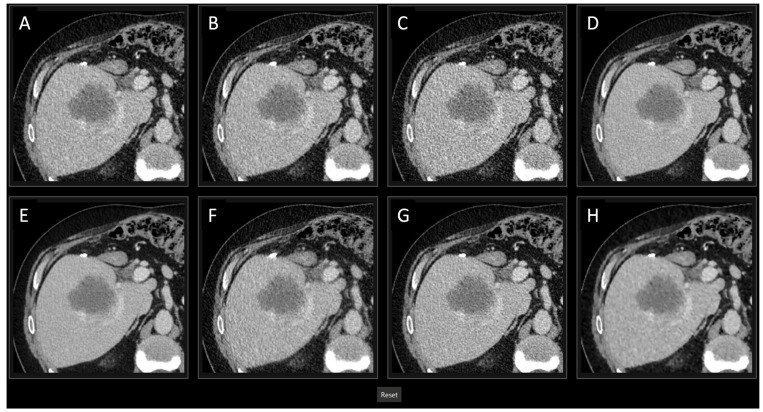
Screenshot of the survey design for the first part of the assessment, with the same region of interest for each reconstruction method. In the first survey, radiologists rated the overall image quality on a scale from 1 (best) to 8 (worst) for various reconstruction methods. The sequence of cases and the arrangement of reconstructions were randomized for each participant and during the evaluation, windowing and zooming were able to be applied. In this case (portal venous), ASIR-V 50% (**A**), PS Soft (**B**), FBP (**C**), DLIR-Medium (**D**), DLIR-High (**E**), PS Ultrasoft (**F**), DLIR-Low (**G**), and ASIR-V 100% (**H**).

In the first part, a total of 20 radiologists (13 junior and 7 senior) with experience ranging from 0 to 17 years blindly ranked the image quality of tumor findings on a scale from 1 (best) to 8 (worst). All findings (28 from the portal venous phase and 5 from the arterial phase) were extracted from consistent ROIs across all reconstruction methods, except for ASIR-V 30% and 70%, and exported in DICOM format to enable windowing during the survey. These ROI displayed the exact same image region for each reconstruction method. To facilitate this process, software was developed using QML and the Qt framework (version 5.1), which presented the ROI for the eight image reconstruction methods in a randomized order, allowing them to be ranked from best to worst, as presented in Figure 2.

#### 2.5.2. Second Part

**Figure 3:**
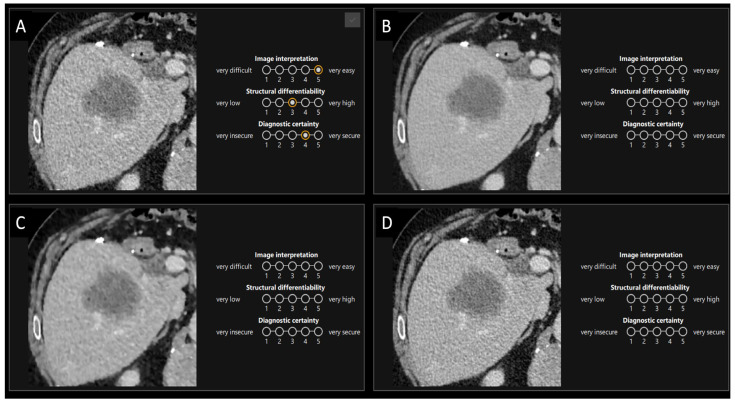
Screenshot of the survey design for the second part of the assessment, with the same region of interest for each reconstruction method. In the second survey, radiologists evaluated image interpretation, structural differentiability, and diagnostic certainty using a 5-point Likert scale for selected reconstruction methods in both the portal venous and arterial phases. The sequence of cases and the arrangement of reconstructions were randomized for each participant and during the evaluation, windowing and zooming were able to be applied. In this case (portal venous), ASIR-V 50% (**A**), DLIR-High (**B**), ASIR-V 100% (**C**) and DLIR-Low (**D**).

In the second part of the survey, three criteria (image interpretation, structural differentiability, diagnostic certainty) of tumor findings were assessed by 24 radiologists (19 junior and 5 senior) with experience ranging from 0 to 15 years. Based on the results from the first survey, four reconstruction methods (ASIR-V 50%, ASIR-V 100%, DLIR-Low, DLIR-High) were selected for further evaluation. Consistent ROI of the same 33 findings in both the portal venous phase and arterial phase were assessed using a 5-point Likert scale, as seen in Figure 3. The presentation of these methods was randomized and blind, with all four reconstruction methods for each contrast agent phase displayed simultaneously.

### 2.6. Statistical Analysis

To determine radiologists’ preferences, the results for each reconstruction type and criterion were averaged for each radiologist. Statistical analyses were performed using the Mann–Whitney U test, as some of the data (rankings and Likert scale) did not meet the assumption of normal distribution, as confirmed by the Shapiro–Wilk test. The initial significance level was set at *p* < 0.05. For both parts of the survey, data were grouped by radiologist experience (junior vs. senior), and differences between these groups were assessed separately for each part. In the first part of the survey, pairwise comparisons were made between eight image reconstruction methods, with a total of 28 comparisons based on the averaged rankings of 20 radiologists. A Bonferroni correction was applied to account for multiple comparisons, resulting in an adjusted significance threshold of α = 0.002. In the second part of the survey, pairwise comparisons were performed for three criteria across four image reconstruction methods for each phase (portal venous and arterial). Since each phase is based on its own set of images, pairwise comparisons were conducted separately for each phase, resulting in 18 comparisons per phase. After applying the Bonferroni correction, the adjusted significance threshold was α = 0.003. All statistical calculations were performed using Python’s SciPy library.

## 3. Results

### 3.1. Quantitative Evaluation

**Figure 4:**
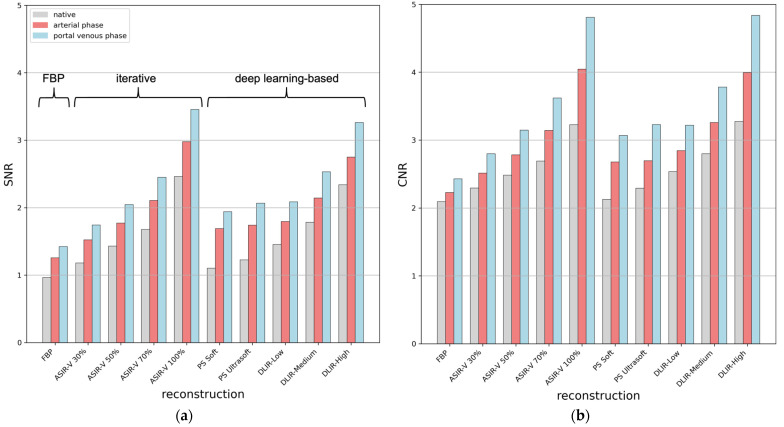
Bar charts showing the mean (**a**) signal-to-noise ratio (SNR) and (**b**) contrast-to-noise ratio (CNR) between the tumor and the directly surrounding tissue for 10 different CT reconstruction techniques across 3 phases (native, arterial, and portal venous) with a slice thickness of 0.625 mm.

Figure 4 displays the SNR and CNR values across 33 findings in the native, arterial, and portal venous phases, highlighting the effectiveness of different CT reconstruction techniques in enhancing image clarity and reducing noise.

The portal venous phase consistently achieved the highest SNR and CNR, followed by the arterial phase, with the native phase showing the lowest values. The data reveal clear trends: both iterative and DL-based methods show significant improvements in SNR and CNR as reconstruction strength increases (see Table 2). When compared to the traditional FBP method across all native, arterial, and portal venous phases, iterative reconstructions improve SNR by 45 ± 4% (ASIR-V 50%) and 145 ± 10% (ASIR-V 100%), while DL-based methods show SNR increases of 28 ± 12% (PS Soft), 37 ± 9% (PS Ultrasoft), 47 ± 5% (DLIR-Low), and 131 ± 13% (DLIR-High). Similar improvements are observed in CNR, with iterative techniques achieving 24 ± 5% (ASIR-V 50%) and 78 ± 22% (ASIR-V 100%), while DL methods show increases of 16 ± 13% (PS Soft), 21 ± 12% (PS Ultrasoft), 27 ± 5% (DLIR-Low), and 78 ± 21% (DLIR-High).

The mean CTDIvol was 6.77 ± 2.96 mGy in the native phase, 6.88 ± 3.46 mGy in the arterial phase, and 7.22 ± 3.55 mGy in the portal venous phase. The corresponding DLP values were 353.67 ± 170.59 mGy·cm, 354.11 ± 187.29 mGy·cm, and 367.94 ± 200.89 mGy·cm, respectively. Differences in CTDIvol directly impact SNR and CNR, potentially explaining variations in image quality across phases.

### 3.2. Qualitative Evaluation

**Figure 5:**
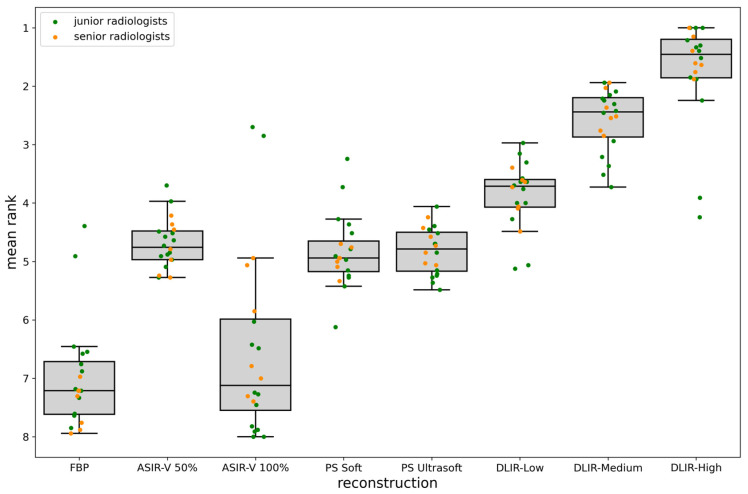
Boxplots showing the average rankings for the eight different reconstruction methods. Additionally, the average rank for each reconstruction type is displayed for each junior radiologist (green dots) and senior radiologist (yellow dots), based on the 33 tumor findings.

In the first part of the survey, DLIR-High was the preferred reconstruction method, with a mean rank of 1.72 ± 0.88, followed by DLIR-Medium (2.58 ± 0.53) and DLIR-Low (3.86 ± 0.56). ASIR-V 50% had a mean rank of 4.69 ± 0.42, while PS Soft and Ultrasoft were ranked similarly (4.84 ± 0.62 and 4.82 ± 0.40, respectively). FBP and ASIR-V 100% had the lowest ranks, 6.98 ± 0.92 and 6.52 ± 1.57, respectively. For the distribution of these ranks, refer to Figure 5. All tumor findings were visible with DL-based reconstructions. The pairwise comparisons of the reconstruction methods using the Mann–Whitney U test indicated no significant differences between FBP and ASIR-V 100%, or between PS Ultrasoft, PS Soft, and ASIR-V 50% (Table 3).

**Figure 6:**
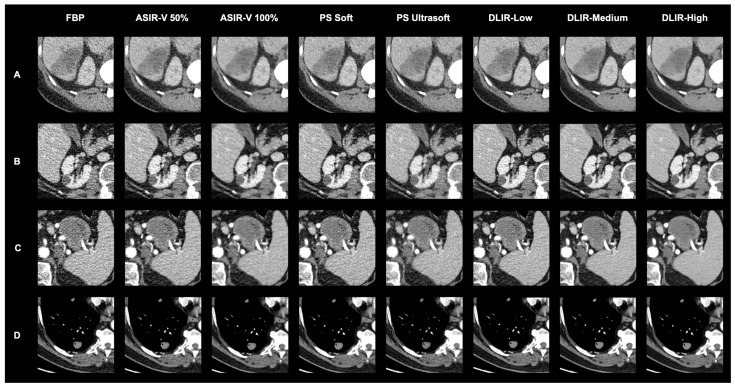
Visual comparison of reconstructed images using different techniques across various tumor types: (**A**) Liver metastasis, (**B**) renal cyst, (**C**) interaortocaval lymph node metastasis, (**D**) lung carcinoma. Findings (**A**,**B**) are shown in the portal venous phase, while findings (**C**,**D**) are presented in the arterial phase. This figure complements the radiological assessments in Figure 5, visually illustrating the differences in image quality observed by the radiologists.

As demonstrated in Figure 5, the radiologists’ evaluations highlighted clear differences in their preferred reconstruction technique. These subjective assessments correlate closely with the objective differences in image quality illustrated in Figure 6. By visually comparing reconstructed images for various tumor types, Figure 6 highlights how the choice of reconstruction technique impacts the clarity and contrast of diagnostic images, further supporting the radiologists’ preferences.

**Figure 7:**
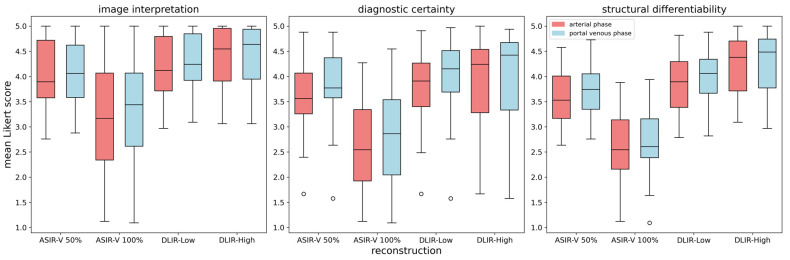
Boxplots of average ratings for four different reconstruction methods across two contrast phases for three criteria.

In the second part of the survey, each of the 33 findings was assessed in both the arterial and portal venous phases using a Likert scale (1 = worst, 5 = best). DLIR-High was rated the highest across both phases for image interpretation, diagnostic certainty, and structural differentiability (mean score: 4.21 ± 0.95), followed by DLIR-Low (4.00 ± 0.91) and ASIR-V 50% (3.78 ± 0.96). ASIR-V 100% received the lowest score (2.91 ± 0.17), as shown in Figure 7. *p*-values for each criterion and contrast phase are shown in Table 4.

The portal venous phase was not significantly preferred over the arterial phase (*p* > 0.19), with average scores of 3.78 ± 0.99 versus 3.67 ± 1.01. However, 24 of 33 findings were rated higher in the portal venous phase, while 9 findings were rated similarly in both phases. In both parts of the survey, no statistically significant difference was observed between junior and senior radiologists (*p*-values: 0.813 and 0.837).

## 4. Discussion

This study evaluated CT reconstruction methods using a blinded procedure involving 28 radiologists with varying experience levels. It focused on how image quality impacts diagnostic performance in tumor staging for up to 33 findings across two contrast phases. The results indicated a clear preference for DLIR among most radiologists, regardless of their experience or the contrast phase, with DLIR-High receiving the highest ratings for image interpretation, structural differentiability, and diagnostic certainty. Junior radiologists showed more variability in their assessments, likely due to their limited experience with CT images. The variability in ratings for ASIR-V 100% may be attributed to the “plastic-like” appearance of images produced by IR algorithms [10,11,12], which has been noted to divide opinions among radiologists. This highlights that applying IR techniques at higher iteration levels is not the right approach—in contrast to DLIR, which maintains the texture of noise, preserving image quality and spatial resolution [14]. This suggests that DL-based reconstruction methods may already outperform other methods, though further improvements are expected as technology advances in the coming years. However, the application of DL-based reconstruction methods does not automatically guarantee better results, as evidenced by the comparison between DLIR and PS.

Table 5 provides an overview of a representative selection of relevant previous studies [3,4,5,6,7,8,9]. These studies have highlighted the superior image quality of DL-based reconstruction methods. However, these studies often focused solely on the images themselves and rarely assessed the visibility of diagnostic findings. In contrast, our study emphasizes qualitative assessments by a diverse group of radiologists, exploring how professional experience affects evaluations and how image quality influences diagnostic performance, making it nearly unique in its approach. In the study by Tamura et al. [7], a different DL algorithm for liver tumor staging was investigated, and once again, DL proved superior to IR in terms of sharpness and diagnostic acceptability. However, comparing this study to others is challenging due to significant variations among different DL methods and the fact that many are tied to specific CT scanners. Xu et al. [9] used virtual monoenergetic images (VMI) at 74 keV from dual-energy CT scans, where a gemstone spectral imaging (GSI) detector captures raw data during a continuous energy shift between low (80 kVp) and high (140 kVp) voltages. This generates two datasets with varying attenuation values, allowing for the reconstruction of material decomposition images and VMI [16]. Dual-energy CT scans also utilize DLIR, but the model has been trained with different data compared to conventional imaging. Thin slices in CT reconstruction provide improved resolution and detail over thick slices, which is particularly beneficial for detecting early-stage lung tumors [17]. This is crucial for tumor staging evaluations, as the early detection of small tumors or metastases enhances staging accuracy and enables more precise therapy planning. Therefore, this study, along with previous studies [5,6,8,9], examined thin slices of 0.625 mm.

In our study, we compared two DL methods and revealed a clear advantage of DLIR over PS. This advantage is primarily due to differing training approaches, because the effectiveness of DL algorithms depends on both the model and the quality of training data [13]: PS combines lower-dose FBP, hybrid IR, and model-based IR images, using routine-dose FBP images as ground truth. In contrast, DLIR utilized lower-dose sinograms from both phantoms and patients, employing a convolutional neural network (CNN) to estimate images against higher-dose FBP images. Generally, a CNN [18] benefits from a larger training dataset, which suggests that further improvements in performance can be expected in the coming years. However, the exclusive availability of TrueFidelity DLIR on GE CT scanners is a limitation for widespread clinical use. In contrast, Pixelshine can be universally retrofitted to various CT systems, but remains behind DLIR in terms of image quality and diagnostic precision. The choice of the appropriate reconstruction method depends heavily on the clinical requirements and the technical conditions. While DLIR is ideal for precise diagnoses and demanding clinical applications, PS could be a viable solution for clinics with different CT equipment due to its flexibility in retrofitting. These results underline the need to further develop pioneering technologies such as DLIR and expand their availability in order to establish them as the new standard in image reconstruction in the long term.

When comparing the findings of this study with other imaging techniques, similar progress can be seen across various radiological fields. For example, a review by Bonada et al. [19] highlights the application of DL in MRI-based brain tumor segmentation. The review emphasizes that the diagnosis, treatment planning, and follow-up of glioblastomas using MRI, including tumor segmentation, are typically carried out by experienced radiologists. However, this process is time-consuming and prone to variability. DL, as part of artificial intelligence (AI), offers the potential to automate segmentation, increase efficiency, and reduce human error. The increasing clinical relevance of DL across multiple medical disciplines underscores the importance of integrating these technologies into routine practice. Widespread implementation could not only support less experienced doctors but also drive further technical advancements, allowing AI methods to improve through continuous clinical use. Nevertheless, challenges such as technical implementation, data collection, and ethical concerns must be addressed to fully harness the potential of AI-based imaging techniques.

This study has several limitations that should be considered. First, the surveys were conducted exclusively within a single-center setting, which may limit the generalizability of the results to other institutions. Differences in patient populations, radiology workflows and CT systems across institutions could influence the applicability of our findings. Conducting multi-center studies in the future would provide a broader perspective and validate the robustness of our results in diverse clinical environments. Second, while we included radiologists with varying levels of experience, the variability in diagnostic performance between junior and senior radiologists might have introduced bias. Although no significant differences were observed in our analysis, more rigorous stratification by experience levels in future studies could help assess how familiarity with DL-based reconstruction techniques impacts diagnostic outcomes. Third, participants rated their diagnostic confidence but were not required to give specific diagnoses for the images, limiting our ability to correlate perceived confidence with actual diagnostic accuracy. Future research should include diagnostic tasks to assess this relationship and identify potential biases, particularly among less experienced radiologists. This step is crucial to ensure that improved image reconstruction leads to measurable diagnostic improvements. Additionally, the retrospective nature of the study and its reliance on routine clinical datasets may constrain the scope of findings. Prospective studies that integrate DLIR into real-time clinical workflows and measure its impact on diagnostic decision-making would offer more comprehensive insights into its utility and limitations. Moreover, such studies should investigate how DLIR influences broader clinical outcomes, such as reporting time and diagnostic error rates, to determine its value in practice.

## 5. Conclusions

This study comprehensively evaluated the performance of various CT reconstruction techniques in the analysis of 33 tumor findings across different contrast phases. Our results demonstrate that DLIR outperforms conventional IR techniques in terms of image interpretation, structural differentiability, and diagnostic certainty regardless of radiologists’ experience levels or the selected contrast phase. These findings highlight the transformative potential of DLIR in clinical practice, offering a reliable and high-quality alternative to existing reconstruction methods. The superior performance observed in our study suggests that DL-based imaging could set a new benchmark not only for tumor staging but also in other radiological fields. The adoption of DLIR in clinical workflows could improve patient outcomes by providing higher-quality imaging at comparable or reduced radiation doses. However, practical implementation requires addressing challenges such as system compatibility as well as integration costs. The proprietary nature of DLIR systems, such as their exclusivity to certain CT platforms, may also influence accessibility and scalability. Universal solutions like PS could play a complementary role in democratizing these advancements. Collaboration between researchers, clinicians and industry partners is crucial to refine DL-based algorithms, optimize training datasets, and ensure equitable access to this technology worldwide.

## Figures and Tables

**Table 1:** Overview of the cohort.

	Overall (n = 33)	Staging (n = 7)	Restaging (n = 26)
Age [years]	64.09 ± 12.77	68.00 ± 9.42	63.04 ± 13.49
Sex (male/female)	16/17	2/5	14/12
BMI [kg/m^2^]	27.49 ± 6.13	26.19 ± 4.99	27.85 ± 6.46
Malignant	26	5	21
Benign	7	2	5

**Table 2:** Percentage improvement in SNR and CNR values compared to FBP.

	ASIR-V 30%	ASIR-V 50%	ASIR-V 70%	ASIR-V 100%	PS Soft	PS Ultrasoft	DLIR-Low	DLIR-Medium	DLIR-High
improvement compared to FBP [%] native									
SNR	23 ± 12	49 ± 28	75 ± 39	156 ± 89	15 ± 7	28 ± 16	51 ± 27	85 ± 46	144 ± 80
CNR	10 ± 14	19 ± 25	29 ± 36	54 ± 60	2 ± 3	10 ± 12	22 ± 30	34 ± 44	56 ± 69
arterial									
SNR	21 ± 15	40 ± 30	67 ± 54	137 ± 135	34 ± 21	38 ± 26	42 ± 32	70 ± 54	118 ± 98
CNR	13 ± 14	25 ± 27	41 ± 42	81 ± 82	20 ± 21	21 ± 20	27 ± 30	46 ± 49	79 ± 84
portal venous									
SNR	23 ± 13	44 ± 26	73 ± 44	144 ± 98	37 ± 21	46 ± 27	47 ± 29	78 ± 49	130 ± 86
CNR	15 ± 14	30 ± 26	49 ± 42	98 ± 86	26 ± 23	33 ± 27	33 ± 30	56 ± 52	99 ± 98
overall mean with phase variability									
SNR	22 ± 1	45 ± 4	72 ± 4	145 ± 10	28 ± 12	37 ± 9	47 ± 5	78 ± 8	131 ± 13
CNR	12 ± 3	24 ± 5	39 ± 10	78 ± 22	16 ± 13	21 ± 12	27 ± 5	45 ± 11	78 ± 21

**Table 3:** *p*-values and significance levels for evaluated reconstruction methods. The significance threshold was adjusted to α = 0.002 after applying the Bonferroni correction.

*p* Value	FBP	ASIR-V 50%	ASIR-V 100%	PS Soft	PS Ultrasoft	DLIR-Low	DLIR-Medium	DLIR-High
FBP	-	<0.001 Sign.	0.715	<0.001 Sign.	<0.001 Sign.	<0.001 Sign.	<0.001 Sign.	<0.001 Sign.
ASIR-V 50%	<0.001 Sign.	-	<0.001 Sign.	0.218	0.516	<0.001 Sign.	<0.001 Sign.	<0.001 Sign.
ASIR-V 100%	0.715	<0.001 Sign.	-	<0.001 Sign.	<0.001 Sign.	<0.001 Sign.	<0.001 Sign.	<0.001 Sign.
PS Soft	<0.001 Sign.	0.218	<0.001 Sign.	-	0.655	<0.001 Sign.	<0.001 Sign.	<0.001 Sign.
PS Ultrasoft	<0.001 Sign.	0.516	<0.001 Sign.	0.655	-	<0.001 Sign.	<0.001 Sign.	<0.001 Sign.
DLIR-Low	<0.001 Sign.	<0.001 Sign.	<0.001 Sign.	<0.001 Sign.	<0.001 Sign.	-	<0.001 Sign.	<0.001 Sign.
DLIR-Medium	<0.001 Sign.	<0.001 Sign.	<0.001 Sign.	<0.001 Sign.	<0.001 Sign.	<0.001 Sign.	-	<0.001 Sign.
DLIR-High	<0.001 Sign.	<0.001 Sign.	<0.001 Sign.	<0.001 Sign.	<0.001 Sign.	<0.001 Sign.	<0.001 Sign.	-

**Table 4:** *p*-values and significance levels for evaluated criteria using a 5-Point Likert scale. The significance threshold was adjusted to α = 0.003 after applying the Bonferroni correction. Results marked with “(Sign.)” are significant at the original significance level (*p* < 0.05), but would not be considered significant when applying the corrected α.

		Arterial Phase				Portal Venous Phase			
		ASIR-50%	ASIR-100%	DLIR-Low	DLIR-High	ASIR-50%	ASIR-100%	DLIR-Low	DLIR-High
image interpretation	ASIR-50%	-	0.023 (Sign.)	0.397	0.080	-	0.014 (Sign.)	0.327	0.140
	ASIR-100%	0.023 (Sign.)	-	0.005 (Sign.)	<0.001 Sign.	0.014 (Sign.)	-	0.002 Sign.	0.002 Sign.
	DLIR-Low	0.397	0.005 (Sign.)	-	0.211	0.327	0.002 Sign.	-	0.515
	DLIR-High	0.080	<0.001 Sign.	0.211	-	0.140	0.002 Sign.	0.515	-
diagnostic certainty	ASIR-50%	-	0.001 Sign.	0.274	0.036 (Sign.)	-	0.002 Sign.	0.212	0.070
	ASIR-100%	0.001 Sign.	-	<0.001 Sign.	<0.001 Sign.	0.002 Sign.	-	<0.001 Sign.	<0.001 Sign.
	DLIR-Low	0.274	<0.001 Sign.	-	0.051	0.212	<0.001 Sign.	-	0.332
	DLIR-High	0.036 (Sign.)	<0.001 Sign.	0.051	-	0.070	<0.001 Sign.	0.332	-
structural differentiability	ASIR-50%	-	<0.001 Sign.	0.122	0.004 (Sign.)	-	<0.001 Sign.	0.066	0.010 (Sign.)
	ASIR-100%	<0.001 Sign.	-	<0.001 Sign.	<0.001 Sign.	<0.001 Sign.	-	<0.001 Sign.	<0.001 Sign.
	DLIR-Low	0.122	<0.001 Sign.	-	0.051	0.066	<0.001 Sign.	-	0.183
	DLIR-High	0.004 (Sign.)	<0.001 Sign.	0.051	-	0.010 (Sign.)	<0.001 Sign.	0.183	-

**Table 5:** Overview of comparable studies. RADs refer to radiologists with relevant experience (Exp.) in qualitative analysis. CA stands for contrast agent phase, which includes arterial (art.) and portal venous (pv.). Pat. stands for patients.

Author/Year	RADs	Exp. [yrs]	Pat.	Region	CA	Method	Outcome
Kim et al. [3] 2020	2	2, 13	58	Lung, mediastinum, liver staging	no	- GE Revolution with low-dose chest protocol (voltage: 120 kVp, slice thickness: 2.5 mm) - Reconstructions: ASIR-V (30%), DLIR (High, Medium) - Evaluation: qualitative analysis of image contrast, noise and the conspicuity of major structures, including the pulmonary arteries, pulmonary veins, trachea and bronchi, lymph nodes, pleura, and pericardium, with 5-point Likert scale; quantitative analysis of signal, noise, SNR and CNR	- Image noise, image quality, SNR, and CNR improve with DLIR compared to ASIR-V 30%. There is no evaluation of findings.
Park et al. [4] 2021	2	9, 21	37	Vessels, Liver, Psoas muscle	art.	- GE Revolution with CT angiography protocol (voltage: 70 kVp, slice thickness: 2.5 mm) - Reconstructions: ASIR-V (80%, 100%), DLIR (High, Medium, Low) - Evaluation: qualitative analysis of quantum mottle and blurring using a 4-grade system; quantitative analysis of mean attenuation, noise, SNR, CNR, and sharpness (blur metric software)	- Qualitative: Mottle and blurring (best to worst) DLIR-High, DLIR-Medium, ASIR-V 80%, DLIR-Low, ASIR-V 100%. - Quantitative: SNR and CNR (best to worst)—DLIR-High = ASIR-V 100%, ASIR-V 80%, DLIR-Medium, DLIR-Low; sharpness better with DLIR than ASIR-V. There is no evaluation of findings.
Zhang et al. [5] 2022	2	13, 23	20 pediatric	abdominal/chest	no	- GE Revolution with abdominal/chest protocol (voltage: 70–80 kVp, slice thickness 0.625 mm) - Reconstructions: ASIR-V (30%, 70%), DLIR (High, Medium) - Evaluation: qualitative analysis of image quality using a 5-point Likert scale; quantitative analysis of noise, SNR, and CNR with a phantom	- Qualitative: DLIR-High had the highest subjective score (abdominal study); no significant difference in subjective scores among the 4 methods (chest study). - Quantitative: DLIR-High showed greatest noise reduction and SNR improvement (abdominal study); DLIR not effective for optimizing extreme CT values (chest study). There is no evaluation of findings.
Li et al. [6] 2022	2	8, 15	25 pediatric	paranasal sinuses	no	- GE Revolution with paranasal sinus protocol (voltage: 100 kVp, slice thickness 0.625 mm) - Reconstructions: FBP, ASIR-V (30%, 50%), DLIR (High, Medium, Low) - Evaluation: qualitative analysis of image quality (ethmoid sinus and nasal cavity) using a 5-point Likert scale; quantitative analysis of noise, SNR, and CNR	- Qualitative: image quality (best to worst) DLIR-High, DLIR-Medium, DLIR-Low, ASIR-V 50%, ASIR-V 30%, and FBP. - Quantitative: As DLIR and ASIR-V strength increased, noise decreased and SNR and CNR improved; DLIR-High was the best. There is no evaluation of findings.
Tamura et al. [7] 2022	2	3, 13	71	Liver staging (hepatocellular carcinoma)	pv.	- Canon Aquilion ONE PRISM with liver staging protocol (voltage: 120 kVp, slice thickness 3 mm) - Reconstructions: adaptive iterative dose reduction 3D (AIDR 3D), advanced intelligent clear-IQ engine (AiCE) - Evaluation: qualitative analysis of noise, artifacts, sharpness, and diagnostic acceptability using a 5-point Likert scale; quantitative analysis of noise, and CNR	- Qualitative: no significant difference in image noise and artifacts; AiCE superior to AIDR 3D in sharpness and diagnostic acceptability. - Quantitative: AiCE had significantly lower noise and higher CNR than AIDR 3D.
De Santis et al. [8] 2023	2	8, 12	51	coronary angiography	art.	- GE Revolution EVO with coronary angiography protocol (voltage: 80–100 kVp, slice thickness 0.625 mm) - Reconstructions: FBP, ASIR-V (10–100%, 10% increments), DLIR (High, Medium, Low) - Evaluation: qualitative analysis of image quality focused on plaques for ASIR-V 50%, ASIR-V 100% DLIR-High and DLIR-Medium using a 4-point Likert scale; qualitative image quality analysis of coronary artery disease (CAD) with ASIR-V 50% and DLIR-Medium; quantitative analysis of noise, SNR and CNR	- Qualitative: DLIR-Medium had the highest image quality; DLIR-High and ASIR-V 50% were comparable, followed by ASIR-V 100%; DLIR-Medium has a very strong correlation with ASIR-V 50% for diagnosing CAD. - Quantitative: DLIR-High showed the lowest noise, comparable to ASIR-V 100%, and achieved the highest objective quality with SNR and CNR similar to ASIR-V 100%; DLIR-Medium had comparable objective image quality to ASIR-V 80% and 90%.
Xu et al. [9] 2023	2	6, >20	30	abdominal	pv.	- GE Revolution with dual energy abdomen protocol (voltage: 80/140 kVp, slice thickness 0.625 and 2 mm) - Reconstructions: virtual monoenergetic (VM) images of 74 keV for ASIR-V 60% and DLIR-High - Evaluation: qualitative analysis of noise, sharpness, noise texture and overall image quality using a 5-point Likert scale; quantitative image quality analysis of noise, SNR and CNR	- Qualitative: DLIR-High significantly improved image quality, especially in 0.625 mm images, compared to ASIR-V 60%. - Quantitative: DLIR-High significantly reduced image noise and increased SNR and CNR compared to ASIR-V 60%. There is no evaluation of findings.
This study	28	0–17	33	chest, abdomen and pelvis staging	art. and pv.	- GE Revolution with stagging protocol (voltage: 120 kVp, slice thickness 0.625 mm) - Reconstructions: FBP, ASIR-V (30%, 50%, 70%, 100%), Pixelshine (Soft, Ultrasoft), DLIR (High, Medium, Low) - Evaluation: qualitative analysis of ranked image quality on a scale from 1 (best) to 8 (worst); qualitative analysis of image interpretation, structural differentiability, and diagnostic certainty for ASIR-V 50%, ASIR-V 100% DLIR-High and DLIR-Low using a 5-point Likert scale; quantitative analysis of SNR and CNR	- Qualitative: DLIR-High ranked highest, followed by DLIR-Medium and DLIR-Low; ASIR-V 50%, Pixelshine Soft, and Ultrasoft were mid-ranked, with FBP and ASIR-V 100% at the bottom; DLIR-High is preferred for all three criteria (image interpretation, diagnostic certainty, and structural differentiability), followed by DLIR-Low, ASIR-V 50%, and ASIR-V 100%. - No significant difference in evaluation between younger and older radiologists, or between arterial and portal venous phases. - Quantitative: SNR and CNR (best to worst)—DLIR-High = ASIR-V 100%, DLIR-Medium = ASIR-V 70%, DLIR-Low = PS Ultrasoft = ASIR-V 50%, PS Soft, ASIR-V 30%, FBP; DLIR-High and ASIR-V 100% showed the highest SNR- and CNR-values with the lowest noise.

## Data Availability

The original contributions presented in this study are included in the article. Further inquiries can be directed to the corresponding author.
